# Incidence, aetiology and outcome of community-acquired acute kidney injury in medical admissions in Malawi

**DOI:** 10.1186/s12882-017-0446-4

**Published:** 2017-01-14

**Authors:** Rhys D. R. Evans, Ulla Hemmilä, Alison Craik, Mwayi Mtekateka, Fergus Hamilton, Zuze Kawale, Christopher J. Kirwan, Hamish Dobbie, Gavin Dreyer

**Affiliations:** 1 Department of Medicine, College of Medicine, Blantyre, Malawi; 2 Department of Nephrology, Queen Elizabeth Central Hospital (QECH), Blantyre, Malawi; 3 Department of Renal Medicine and Transplantation, Bart’s Health, London, UK; 4 University College London Centre for Nephrology, Royal Free Hospital, London, UK

**Keywords:** Acute kidney injury, Epidemiology, Haemodialysis, HIV, Nephrotoxicity, Sepsis

## Abstract

**Background:**

Epidemiological data on Acute Kidney Injury (AKI) from low-income countries is sparse. The aim of this study was to establish the incidence, severity, aetiology, and outcomes of community-acquired AKI in Malawi.

**Methods:**

We conducted a prospective observational study of general medical admissions to a tertiary hospital in Blantyre between 27^th^ April and 17^th^ July 2015. All patients were screened on admission with a serum creatinine; those with creatinine above laboratory reference range were managed by the nephrology team. Hospital outcome was recorded in all patients.

**Results:**

Eight hundred ninety*-*two patients were included; 188 (21 · 1%) had kidney disease on admission, including 153 (17 · 2%) with AKI (median age 41 years; 58 · 8% HIV seropositive). 60 · 8% of AKI was stage 3. The primary causes of AKI were sepsis and hypovolaemia in 133 (86 · 9%) cases, most commonly gastroenteritis (*n* = 29; 19 · 0%) and tuberculosis (*n* = 18; 11 · 8%). AKI was multifactorial in 117 (76 · 5%) patients; nephrotoxins were implicated in 110 (71 · 9%). Inpatient mortality was 44 · 4% in patients with AKI and 13 · 9% if no kidney disease (*p* <0.0001). 63 · 2% of patients who recovered kidney function left hospital with persistent kidney injury.

**Conclusion:**

AKI incidence is 17 · 2% in medical admissions in Malawi, the majority is severe, and AKI leads to significantly increased in-hospital mortality. The predominant causes are infection and toxin related, both potentially avoidable and treatable relatively simply. Effective interventions are urgently required to reduce preventable young deaths from AKI in this part of the world.

**Electronic supplementary material:**

The online version of this article (doi:10.1186/s12882-017-0446-4) contains supplementary material, which is available to authorized users.

## Background

Acute Kidney Injury (AKI) refers to a rapid worsening or loss of kidney function caused by a variety of different mechanisms. Most epidemiological data on AKI comes from developed world settings. Here, AKI is predominantly hospital acquired, affecting up to 22% of adult patients during an inpatient stay [1, 2]. It leads to adverse outcomes for individuals, even in its mildest forms, with mortality across all stages of AKI estimated at 21%, increasing with AKI severity [2, 3]. Moreover, it can lead to other organ dysfunction and the development or progression of chronic kidney disease (CKD) [4].

Whilst unknown, the potential global burden of AKI is vast. 85% of the world’s population lives in developing world settings and assuming the same incidence and outcome of AKI to high-income countries, this equates to an estimated 13 · 3 million cases of AKI per year worldwide, resulting in a potential 1 · 7 million deaths [5]. The greatest impact of AKI may therefore be in the poorest parts of the world. It is in these regions, however, where epidemiological data on AKI is most limited and where adverse outcomes from AKI may be preventable with relatively cheap and simple interventions [6, 7].

The International Society of Nephrology (ISN) launched the 0by25 initiative in response to this concern about a significant burden of undiagnosed and untreated AKI in resource poor areas [2]. The initiative aims to eliminate preventable deaths from AKI by 2025. One of its key initial goals is to collect epidemiological data on AKI and its outcomes, especially in low-income settings. This is an essential first step in attempts to improve management of AKI and its consequences worldwide.

The aim of this study was to determine the incidence, severity, aetiology, and outcomes of community-acquired AKI (com-AKI) in general medical admissions at a tertiary hospital in Malawi, a resource-poor country in sub-Sahara Africa.

## Methods

### Study design and setting

We conducted a prospective, observational study at Queen Elizabeth Central Hospital (QECH), Blantyre, Malawi. Government healthcare, although resource-limited, is free at the point of delivery, including widespread access to antiretroviral therapy (ART) for patients with HIV. Blantyre is the second largest city in Malawi and QECH acts as both a district and a regional hospital, although the majority of patients admitted are from Blantyre district itself, population approximately 1 million [8]. Around 15 medical patients are admitted per day and medications to manage common causes of sepsis (broad spectrum antibiotics, anti-malarials and anti-tuberculous drugs) are usually available. QECH acts as the nephrology referral centre for the southern region including free provision of haemodialysis to patients with AKI and to a limited cohort with end stage kidney disease.

### Participants

Patients were enrolled between a pre-specified consecutive 12-week time period, 27^th^ April-17^th^ July 2015. All medical admissions ≥14 years (local age cut off for admission under adult medicine) were included. Patients unable to consent or transferred to the medical service from another specialty ward or hospital were excluded.

### Data collection

Baseline demographic and clinical data were collected in all patients and screening for AKI undertaken with a serum creatinine. Data was obtained from the patient’s medical record; the admission diagnosis was that documented by the admitting physician.

Patients with an admission creatinine above the laboratory reference range (>90 μmol/L in women; >104 μmol/L in men) were reviewed daily and managed by the nephrology team. Urine output was recorded and the creatinine was reassessed at 24 h (day 1) and then every 48 h until death in hospital or discharge. We documented the nature and most likely cause of renal impairment based on the assessment of all available clinical and laboratory investigations as well as details of management thereafter.

Patients with a normal admission creatinine were managed by general medical physicians and not routinely seen by the study team. These patients did not have creatinine repeated by the study team and the development of AKI in hospital was not assessed. Length of stay and hospital outcome (discharge or death) was recorded in all patients. If a patient was admitted more than once during the study period, each admission was treated as a new case.

### Definition of AKI

AKI and its staging, Acute Kidney Disease (AKD) without AKI, and CKD were defined by Kidney Disease Improving Global Outcomes (KDIGO) criteria (Additional file 1 Table S1). Patients not fulfilling these criteria were deemed as having no kidney disease (NKD). Community-acquired AKI (com-AKI) is AKI that is detected at, or within 48 h, of hospital admission.

AKI aetiology was categorized by Acute Kidney Injury Network (AKIN) criteria: Sepsis and hypoperfusion, Toxins, Obstruction, and Parenchymal kidney disease (STOP) [9]. The discharge creatinine was the last creatinine measured as an inpatient. Renal recovery was pre-defined by our own definition as partial if the discharge creatinine was >10% below the peak value, and complete if within the laboratory reference range, or within 10% of a previously known baseline value.

### Creatinine measurement

Creatinine was measured by Jaffe method [either by Flexor Junior Clinical Chemistry Analyzer (Vital Scientific, Dieren, The Netherlands) or by Mindray Chemistry Analyzer BS-120 (Shenzen Mindray Bio-Medical Electronics Company, Shenzen, China)] in a local laboratory. A second analyser was used due to unresolvable technical problems with the original machine during part of the study. Both machines were calibrated according to the manufacturer’s instructions.

### Outcome measures

Primary outcome measures were the incidence proportion of com-AKI, the aetiology of AKI, and the effect of AKI on in-hospital mortality. Secondary outcomes were the severity of AKI, an evaluation of the independent risk factors for the presence of AKI on admission and death from AKI, in addition to an assessment of renal recovery from AKI. Given the high prevalence of HIV in medical admissions, we also evaluated the aetiology and outcomes from AKI in patients with HIV.

### Statistical analysis

We compared variables between patients with NKD, any stage of AKI, stage 1 or 2 AKI and stage 3 AKI using standard parametric, non-parametric and Chi^2^ tests according to the distribution of variables. We combined patients with stage 1 or 2 AKI into a single group, given the relatively small number of patients with less severe AKI.

We performed a survival analysis, comparing patients with NKD, stage 1 or 2 AKI and stage 3 AKI, using the log rank test to examine for differences in survival between these groups. This analysis was censored at 15 days since the number at risk in each group reduced dramatically at this point (<10 in both AKI groups). We calculated crude and adjusted hazard ratios for mortality, adjusting for a priori confounding variables of age (< or >40 years) and sex, as well as variables associated with AKI development and mortality in univariate analyses with a pre-defined *p* value for addition into the model of <0 · 05. For this analysis, we excluded patients who were alive and still inpatients at the end of the pre-specified study period.

We used a logistic regression model with forward fitting of variables to identify the independent risk factors for any stage of AKI on admission including in the model risk factors most strongly associated with AKI in a univariate analysis with a *p* value of <0 · 05 for entry in to the model. In a subgroup analysis, we compared patients with AKI who were HIV positive to those who were HIV negative or unknown.

All analyses were conducted using Stata v 10 with a *p* value of <0 · 05 considered to be statistically significant.

## Results

### Participants

**Fig. 1:**
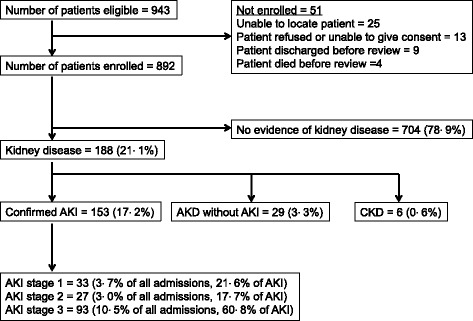
Patient enrollment, incidence and severity of AKI

943 patients were admitted under general medicine during the study period and 892 were enrolled (Fig. 1).

### Descriptive data

Baseline characteristics are outlined in Table 1. The median age was 37 years (interquartile range [IQR] 30–52), 391 (43 · 8%) patients had HIV and 109 (12 · 2%) had pre-existing hypertension, but other co-morbidities were relatively uncommon. 324 (36 · 3%) patients were taking ART, 311 (34 · 9%) tenofovir (TDF) based.

**Table 1:** Baseline data in all patients in the study population, in patients with no kidney disease (NKD), and in patients with Acute Kidney Injury (AKI). Variables are compared between NKD and AKI

Demographic Variable	All patients *N* = 892	No Kidney Disease (NKD) *N* = 704	Acute Kidney Injury (AKI) *N* = 153	*P* value
Age (median; IQR)	37 (30–52)	36 (28–50)	41 (32–58)	0 · 0008
Male sex	500 (56 · 1%)	388 (55 · 1%)	92 (60 · 1%)	0 · 257
Medical History				
HIV	391 (43 · 8%)	281 (39 · 9%)	90 (58 · 8%)	<0 · 0001
HTN	109 (12 · 2%)	78 (11 · 1%)	24 (15 · 7%)	0 · 112
CVD^a^	52 (5 · 8%)	39 (5 · 6%)	12 (7 · 8%)	0 · 277
TB	43 (4 · 8%)	28 (4 · 0%)	15 (9 · 8%)	0 · 003
DM	34 (3 · 8%)	20 (2 · 9%)	10 (6 · 5%)	0 · 025
Liver Disease	8 (0 · 9%)	5 (0 · 7%)	3 (2 · 0%)	0 · 145
Malignancy	7 (0 · 8%)	3 (0 · 4%)	3 (2 · 0%)	0 · 48
Known CKD	5 (0 · 6%)	0 (0%)	2 (1 · 3%)	0 · 027
Medications				
NSAID	339 (38 · 1%)	259 (37 · 0%)	66 (43 · 1%)	0 · 153
TDF ART	311 (34 · 9%)	234 (33 · 2%)	66 (43 · 1%)	0 · 02
Diuretic	144 (16 · 2%)	109 (15 · 5%)	26 (17 · 0%)	0 · 652
ACEi or ARB	31 (3 · 5%)	19 (2 · 7%)	6 (3 · 9%)	0 · 419
Non TDF ART	13 (1 · 5%)	3 (0 · 4%)	6 (3 · 9%)	0 · 001
Admission Diagnosis				
LRTI	215 (24 · 1%)	196 (27 · 8%)	16 (10 · 4%)	<0 · 0001
Sepsis^b^ unclear source	104 (11 · 6%)	81 (11 · 5%)	21 (13 · 7%)	0 · 442
GE	79 (8 · 9%)	46 (6 · 5%)	30 (19 · 6%)	<0 · 0001
Meningitis	66 (7 · 4%)	61 (8 · 7%)	5 (3 · 3%)	0 · 023
Stroke	63 (7 · 1%)	51 (7 · 2%)	8 (5 · 2%)	0 · 37
Heart Failure	62 (7 · 0%)	45 (6 · 4%)	13 (8 · 5%)	0 · 348
Symptomatic Anaemia	60 (6 · 7%)	45 (6 · 4%)	11 (7 · 2%)	0 · 718
TB	43 (4 · 8%)	28 (4 · 0%)	15 (9 · 8%)	0 · 003
Malaria	33 (3 · 7%)	21 (3 · 0%)	9 (5 · 9%)	0 · 077
Liver failure	26 (2 · 9%)	14 (2 · 0%)	11 (7 · 2%)	0 · 001
Disseminated malignancy	17 (1 · 9%)	14 (2 · 0%)	3 (2 · 0%)	0 · 982
Other	124 (13 · 9%)	102 (14 · 5%)	11 (7 · 2%)	0 · 45

*AKI* acute kidney injury, *HIV* human immunodeficiency virus, *HTN* hypertension, *CVD* cardiovascular disease, *TB* tuberculosis, *DM* diabetes mellitus *CKD* chronic kidney disease, *NSAID* non steroidal anti-inflammatory drug, *TDF ART* tenofovir based antiretroviral therapy, *ACEi* angiotensin converting enzyme inhibitor, *ARB* angiotensin receptor blocker, *LRTI* lower respiratory tract infection (including pleural effusion), *GE* gastroenteritis

^a^ischaemic heart disease, cerebrovascular disease, or peripheral vascular disease

^b^clinical diagnosis

Increased age, HIV seropositivity, a history of previous tuberculosis (TB), diabetes melllitus (DM), or known CKD, and TDF use were all more prevalent in patients with any stage of AKI. Most admissions were due to infective illnesses. An admission diagnosis of gastroenteritis (GE), acute TB, and liver failure were significantly more common in patients with AKI (Table 1).

### Outcome data

#### AKI Incidence proportion in the study population

Of 892 patients enrolled, 188 (21 · 1%) had evidence of kidney disease (Fig. 1). 153 (17 · 2%) patients had AKI, 29 (3 · 3%) had AKD without AKI, and 6 patients (0 · 6%) had established stable CKD. Of those with AKI, the maximum stage was stage 1 in 33 (21 · 6%) patients, stage 2 in 27 (17 · 7%) patients, and stage 3 in 93 (60 · 8%) patients. The median presenting creatinine values in patients with Stage 1,2 and 3 AKI was 140 μmol/L (124–159), 212 μmol/L (157–297), and 565 μmol/L (327–878) respectively.

51 (6 · 0%) patients (9 AKI; 42 NKD) remained in hospital at the end of the study and were excluded from the outcome analysis.

#### AKI Aetiology

The primary causes of AKI are outlined in Table 2. In 117 (76 · 5%) patients, AKI was multifactorial. Nephrotoxins as a contributing factor, as opposed to the primary cause, were implicated in 110 (71 · 9%) cases of AKI. The most common nephrotoxins implicated were tenofovir based antiretroviral therapy (TDF ART) (*n* = 61; 40 · 0%) and non-steroidal anti-inflammatory drugs (NSAIDs) (*n* = 53; 34 · 6%). Traditional or herbal remedies had been taken by 12 (7 · 8%) patients with AKI prior to admission.

**Table 2:** Primary Causes of Acute Kidney Injury (AKI) in patients with any stage of AKI, stage 1 or 2 AKI, and stage 3 AKI

Primary cause of AKI	AKI all (*n* = 153)	AKI stage 1 or 2 (*n* = 60)	AKI stage 3 (*n* = 93)
Sepsis and hypoperfusion	133 (86 · 9%)	55 (91 · 7%)	78 (83 · 9%)
GE^a^	29 (19 · 0%)	12 (20 · 0%)	17 (18 · 3%)
TB	18 (11 · 8%)	5 (8 · 3%)	13 (14 · 0%)
Heart failure	16 (10 · 5%)	10 (16 · 7%)	6 (6 · 5%)
Malaria	12 (7 · 8%)	4 (6 · 7%)	8 (8 · 6%)
Dehydration of other cause	12 (7 · 8%)	6 (10 · 0%)	6 (6 · 5%)
LRTI	10 (6 · 5%)	6 (10 · 0%)	4 (4 · 3%)
Sepsis of unclear source	9 (5 · 9%)	2 (3 · 3%)	7 (7 · 5%)
Liver Failure	8 (5 · 2%)	3 (5 · 0%)	5 (5 · 4%)
Meningitis	7 (4 · 6%)	4 (6 · 7%)	3 (3 · 2%)
Sepsis of other known source	4 (2 · 6%)	1 (1 · 7%)	3 (3 · 2%)
UTI	1 (0 · 7%)	0 (0 · 0%)	1 (1 · 1%)
Other	7 (4 · 6%)	2 (3 · 3%)	5 (5 · 4%)
Toxin (as primary cause)	5 (3 · 3%)	3 (5 · 0%)	2 (2 · 1%)
NSAID	4 (2 · 6%)	3 (5 · 0%)	1 (1 · 1%)
ACEi or ARB	1 (0 · 7%)	0 (0 · 0%)	1 (1 · 1%)
Urinary tract Obstruction	7 (4 · 6%)	1 (1 · 6%)	6 (6 · 5%)
Prostate disease	2 (1 · 3%)	0 (0 · 0%)	2 (2 · 2%)
Cervical malignancy	1 (0 · 7%)	0 (0 · 0%)	1 (1 · 1%)
Bladder Malignancy	1 (0 · 7%)	0 (0 · 0%)	1 (1 · 1%)
Unclear/other	3 (2 · 0%)	1 (1 · 7%)	2 (2 · 2%)
Parenchymal Kidney disease	4 (2 · 6%)	0 (0 · 0%)	4 (4 · 3%)
Acute GN	3 (2 · 0%)	0 (0 · 0%)	3 (3 · 3%)
TIN (presumed)	1 (0 · 7%)	0 (0 · 0%)	1 (1 · 1%)
Unclear	4 (2 · 6%)	1 (1 · 7%)	3 (3 · 2%)

*AKI* acute kidney injury, *GE* gastroenteritis, *TB* tuberculosis, *LRTI* lower respiratory tract infection (including pleural effusion), *UTI* urinary tract infection, *NSAID* non steroidal anti-inflammatory drug, *ACEi* angiotensin converting enzyme inhibitor, *ARB* angiotensin receptor blocker, *TDF ART* tenofovir based antiretroviral therapy, *GN* glomerulonephritis, *TIN* tubulointerstitial nephritis

^a^including typhoid and non-typhoidal salmonella

#### Cases of AKI treated with haemodialysis

Renal replacement therapy was indicated in 21 (13 · 7%) patients with AKI; acute haemodialysis was provided in 8 (38 · 1%). Reasons for not providing dialysis were clinician judgement of medical futility (*n* = 9), haemodynamic instability precluding haemodialysis (*n* = 3), and logistical reasons (*n* = 1). The primary causes of AKI in patients that underwent haemodialysis were: falciparum malaria (*n* = 4), post-infective (malaria) glomerulonephritis (*n* = 1), typhoid (*n* = 1), non-typhoid salmonella (*n* = 1), and multiple drug toxicity (*n* = 1). Median number of sessions was 4 (3 · 5–6). Renal recovery (dialysis independence) occurred in 6 (75%) patients, dialysis was withdrawn in one patient, and one patient died while being actively treated.

#### In-hospital mortality

Crude inpatient mortality was 44 · 4% (*n* = 64) in patients with AKI compared with 13 · 9% (*n* = 92) in patients with no kidney disease (*p* <0 · 0001). Crude mortality in patients with AKI was higher with increasing AKI severity [*n* = 18 (30 · 0%) in stage 1 and 2 vs. *n* = 46 (49 · 5%) in stage 3; *p* = 0 · 017].

**Fig. 2:**
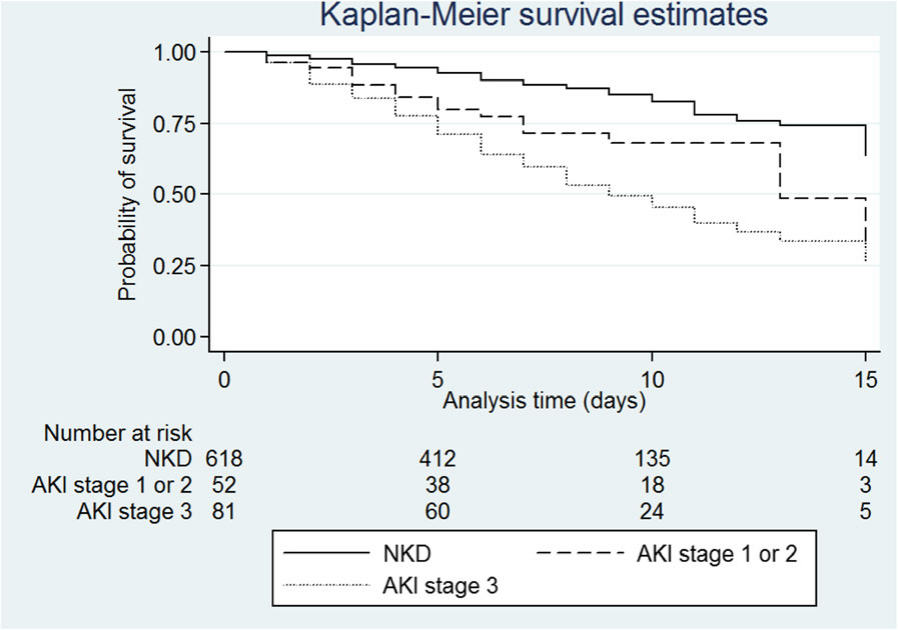
Kaplan-Meier analysis demonstrating survival probability in patients with No Kidney Disease (NKD), AKI stage 1 or 2, and AKI stage 3 up to 15 days. 137 (87 · 8%) of 156 deaths occurred before or on day 15. Patients have been censored for hospital discharge, as follow up was not undertaken after discharge to assess for out of hospital mortality

In a survival analysis, patients with stage 3 AKI had significantly higher in-hospital mortality than patients with either NKD or stage 1 or 2 AKI (chi^2^ test 59.4; log rank test *p* <0 · 0001, Fig. 2). Crude hazard ratios (95% confidence interval) for mortality comparing patients with stage 1 or 2 AKI and stage 3 AKI to patients with NKD were 2 · 33 (1 · 40–3 · 87, *p* = 0 · 001) and 3 · 68 (2 · 57–5 · 27, *p* <0 · 0001) respectively. Hazard ratios for mortality compared to NKD adjusted for age, sex, use of any toxin and HIV status were 2 · 14 (1 · 27–3 · 59, *p* = 0 · 004) for stage 1 or 2 AKI and 3 · 37 (2 · 32–4 · 89, *p* <0 · 0001) for stage 3 AKI.

***Independent risk factors for developing any stage of AKI*** *were* age >40 years (*p* = 0 · 0001), HIV seropositivity (*p* <0 · 0001), underlying DM (*p* = 0 · 01), presentation with GE (*p* <0 · 0001), liver failure (*p* = 0 · 0002) or TB (*p* = 0 · 0034), and the use of any nephrotoxin prior to admission (*p* = 0 · 04).

***Independent risk factors for in-hospital mortality in patients with AKI*** *were* stage of AKI (stage 3 vs. stage 1 or 2; *p* = 0 · 016), age >40 (*p* = 0 · 02), and use of any nephrotoxin (*p* = 0 · 04).

#### Renal recovery

Some degree of renal recovery was evident by discharge in 68 (47 · 2%) patients with AKI. Any degree of renal recovery was more likely in stage 1 and 2 compared to stage 3 AKI [*n* = 35 (65 · 0%) vs. *n* = 33 (38 · 8%), *p* = 0 · 002]. If renal recovery occurred, persistent kidney injury was present at discharge in 43 (63 · 2%) AKI patients overall, 17 (48 · 6%) if AKI stage 1 or 2, and 26 (78 · 0%) if AKI stage 3 (*p* = 0 · 01). The median creatinine in AKI survivors at hospital discharge was 129 μmol/L (114–167), 126 μmol/L (97–215), and 552 μmol/L (324–921) in stages 1,2 and 3 respectively.

Median length of hospital stay was longer in any stage of AKI (7 · 5 days; 5–12) compared with NKD (6 days; 4–10) but this did not reach statistical significance (*p* = 0 · 052).

#### HIV subgroup analysis

The characteristics of patients with AKI comparing HIV status are summarised in Table 3. Patients with HIV were younger. There were no significant differences in AKIN aetiological categories of AKI (Sepsis and hypoperfusion, Toxins, Obstruction and Parenchymal Kidney Disease [STOP]), AKI severity, mortality, length of stay or renal recovery between patients with HIV and those without HIV or in whom HIV status was unknown.

**Table 3:** Comparison of AKI in patients with HIV to patients without HIV or in whom HIV status was unknown

	No HIV or HIV unknown (*n* = 63)	HIV (*n* = 90)	*P* value
Demographics			
Age (median; IQR)	53 (35–71)	38 (32–46)	0 · 0004
Male sex	35 (55 · 6%)	57 (63 · 3%)	0 · 334
Primary cause of AKI			
Sepsis and hypoperfusion	51 (80 · 1%)	82 (91 · 1%)	0 · 058^a^
Toxin	1 (1 · 6%)	4 (4 · 4%)	
Obstruction	6 (9 · 5%)	1 (1 · 1%)	
Parenchymal Kidney disease	2 (3 · 2%)	2 (2 · 2%)	
Unclear	3 (4 · 8%)	1 (1 · 1%)	
AKI Severity			
AKI Stage 3	39 (61 · 9%)	54 (60 · 0%)	0 · 81
Outcomes			
In-hospital mortality	22 (37 · 9%)	42 (48 · 4%)	0 · 20
Length of stay in days (median; IQR)	7 · 5 (4–13)	7 · 5 (5–11)	0 · 74
Renal recovery (any)	27 (49 · 1%)	41 (49 · 4%)	0 · 97
Persistent kidney injury at discharge^b^	19 (70 · 4%)	24 (58 · 4%)	0 · 32

*AKI* acute kidney injury, *HIV* human immunodeficiency virus

^a^Chi^2^ test assessing the association of the aetiological category of AKI between groups

^b^expressed as a proportion of those with any renal recovery

## Discussion

This is the first study to prospectively determine the incidence, aetiology, severity and outcomes of com-AKI in an unselected patient cohort in sub-Sahara Africa (SSA). AKI is common, serious and treatable in the developed world. Estimates of incidence based on developed world data suggest an undiagnosed epidemic in less developed countries, and reports of deficiencies in care raise the concept that young lives are being unnecessarily lost [2]. However, particularly in SSA, data to corroborate these hypotheses are sparse. We therefore undertook this real-life, pragmatic study to understand the current epidemiology of AKI in Malawi.

### Key results

We have demonstrated that com-AKI is both common and severe in medical admissions in Malawi. 17 · 2% of patients had established AKI on admission over the 3-month data collection period; 10 · 5% of general medical admissions had stage 3 AKI. Patients were young, from an economically viable section of the population, and HIV prevalence was high, particularly in patients with AKI.

The predominant aetiological category of AKI was Sepsis/hypoperfusion, encompassing conditions that are amenable to early identification and effective treatments routinely available across much of SSA. The most common infections were GE, acute TB, and falciparum malaria.

Hospital outcomes in patients with AKI were poor and these worsened with AKI stage. Hospital mortality was 44 · 4% in patients with AKI overall and almost half of patients with stage 3 AKI died. The majority of patients with AKI who survived left hospital with persistent and severe renal injury, particularly patients with stage 3 AKI.

### Interpretation

AKI affects 1 in 5 inpatients in developed settings [2], but the majority is mild and hospital-acquired [10]. Com-AKI is less common, with incidence estimates of 4 · 3% in the UK [11], whereas in SSA com-AKI predominates [6, 7, 12].

Rates of com-AKI in this study were more than quadruple what is seen in developed settings (Additional file 2 Table S2). Moreover the kidney injury was predominantly severe (stage 3 in 60 · 5%) as opposed to the mild pattern of AKI seen in developed settings (stage 1 in 80%) [2]. This burden and severity of AKI is likely multifactorial in nature. Delayed referral to hospital due to limited awareness of kidney injury and the resources to detect it in health centres and district hospitals [13, 14], logistical and financial challenges transporting patients to hospital, an admission cohort primed for AKI in terms of co-morbidity (high HIV prevalence), community nephrotoxin use, and severe acute presenting illness all contribute.

Data from SSA with which to compare these incidence and severity estimates is limited [7]. The rate of AKI in the two previous prospective studies in medical cohorts in SSA were similar to ours: 16% in 387 patients presenting with sepsis in urban Uganda and 15 · 2% in 151 medical inpatients in rural Ethiopia [15, 16]. The majority of AKI was severe in both, with 46-48% of AKI stage 3.

The age of patients with AKI in our study is similar to findings from other studies from SSA, where mean ages of patients with AKI have ranged from 28 · 7–44 · 4 years [7]. Patients are around 20 years younger than AKI cohorts in developed settings [10].

The prevalence rate of HIV is approximately 10% in 15–49 year-olds in the general population in Malawi [17]. Patients with HIV were disproportionately represented in the medical admission cohort as a whole, but particularly so if presenting with AKI. 58 · 8% of patients with AKI had HIV; similar rates have been recently reported in other SSA AKI cohorts [15]. HIV may increase the risk of AKI through direct renal injury, through ART nephrotoxicity, and through susceptibility to acute infective illness. Routine measurement of renal function pre or post initiation of TDF ART (first-line in adults) is not recommended in Malawi despite the potential nephrotoxic effects of TDF [18], which may delay diagnosis of AKI in this cohort. Importantly, AKI in Malawi is occurring in patients that are young with limited comorbidity outside HIV. Such patients are often responsible for the social and financial stability of extended families and thereby the impact of AKI extends beyond the individual.

Medical causes of AKI predominate in SSA and, similar to previous data, AKI in this study was largely due to infective illnesses [6, 7]. Microbiological causes of GE were rarely identified, as faecal microbiology is not routinely undertaken. Typhoid and non-typhoid salmonella bacteraemia did, in some cases, present with diarrhoeal illness. Despite this study occurring outside malaria season, it remained a common cause of AKI. Acute decompensated heart failure was the commonest cause of renal hypoperfusion outside infective illness, and this was particularly prevalent in the non-HIV AKI group.

In addition to infection, nephrotoxins (in particular NSAIDs) were the other main contributors to AKI. The crucial significance of these causes and contributors is that it suggests the majority of this AKI is preventable and treatable by relatively simple means such as early fluid resuscitation, treatment of the underlying condition, and avoidance of nephrotoxic drugs. These are universally achievable interventions that don’t require sophisticated diagnostic techniques or expensive treatments. Despite this, deficiencies in resources and skills to manage even these basic aspects of AKI across SSA persist [13, 14].

The worldwide pooled mortality from AKI is 21% [2] and this rises to 32% in studies undertaken in SSA [7]. In-hospital mortality in this study was almost double worldwide estimates. Mortality increased with AKI stage, 49 · 5% in stage 3 AKI, a closer reflection of the worldwide mortality of 42% seen at this stage of disease. In part, the high overall mortality reflects the severity of AKI encountered in this setting but may also relate to lack of resources to manage underlying conditions, in particular severe sepsis.

Some form of renal recovery occurred in the majority of adult survivors although this recovery was largely incomplete. This may represent patients with resolving AKI, undiagnosed or *de novo* CKD, and highlights the need for follow up and assessment of longer-term outcomes post AKI, especially in this part of the world. Recurrent, severe AKI in SSA may contribute to the growing burden of CKD in this setting [19].

Taken together, the mortality and persistent renal injury seen in this study are concerning. Moreover, the outcomes in this study, whilst poor, were achieved in a clinical research environment in which all patients had immediate assessment of renal function, management by a nephrologist, and free access to renal replacement therapy (RRT). This is not the case during routine clinical practice across SSA. The poor outcomes therefore represent, in our view, a “best-case scenario” for SSA and highlight the urgency with which we as a global healthcare community must improve AKI detection and management in resource poor settings worldwide.

#### Strengths and limitations

Most studies investigating AKI in SSA are retrospective case series, often focusing on patients that present requiring RRT or on a single cause of AKI (Additional file 3 Table S3). There has been no previous prospective study in SSA that we are aware of in which an unselected medical admission cohort has been screened for com-AKI. This is important as laboratory assessment of renal function is not routine in SSA and therefore only through this approach can the frequency, severity and outcomes of com-AKI be accurately determined. Our study is the first to do this in SSA, screening 892 patients, and the 153 patients with com-AKI identified represents the largest prospective AKI cohort reported from this region of the world.

This was a single centre study conducted during a single season of the year in medical admissions only. This study focused on community acquired AKI and the development of AKI in hospital was not assessed. Lack of known baseline creatinines in almost all patients and practical challenges in collecting urine output data (e.g. lack of urine measuring jugs, lack of catheter bags with graduations to accurately measure urine volume) may have resulted in some patients with AKI being classified as AKD, underestimating the true incidence. Unresolvable technical problems with the creatinine analyser meant a second machine had to be used for part of the study and consequently no data is available to assess any assay variability between the different machines. It is unlikely this resulted in any significant misclassification of stage of AKI and whilst we recognise that this is a limitation of the study, the situation represents the real-life pragmatic challenges related to research conducted in this and similar settings. Resource restrictions meant supporting diagnostics were limited and causes of AKI were based largely on clinical assessment but this is reflective of standard clinical practice across much of SSA. Hospital outcome was captured in all patients, but longer follow up thereafter was not.

Our aim, and that of the International Society of Nephrology 0by25 Initiative, is to investigate AKI in low-income settings. Malawi ranks as one of the poorest countries in the world [20] and therefore this study reflects AKI in a truly low-income setting. This study was undertaken in a centre which serves both urban and rural populations. Healthcare is provided free at the point of delivery and therefore the population represented in this study is not influenced by financial factors limiting access to care. We are sanguine about the fact that many other neighbouring healthcare systems levy a charge for even basic interventions including intravenous fluids and antibiotics. Thus, our study may not be generalisable to these settings, where outcomes from AKI may be even worse.

## Conclusions

Com-AKI incidence is 17 · 2% in medical admissions in Malawi, the majority is severe, and AKI leads to significantly increased in-hospital mortality. Sepsis, hypovolaemia related to sepsis, and toxins are the predominant causes. As simple treatments are often available, earlier identification of AKI is essential both in hospital and at lower tiers of the healthcare system, and resource should be concentrated on education programmes and point of care tests to improve this. A study of the effect of a simple intervention can then be done to assess its impact on mortality as part of attempts to reduce preventable deaths from AKI in this part of the world.

## Additional files

Additional file 1: Table S1. Definitions – Acute Kidney Injury (AKI), Acute Kidney Disease/Disorder (AKD) without AKI, Chronic Kidney Disease (CKD) and No Kidney Disease (NKD). (DOCX 94 kb)
Additional file 2: Table S2. Comparison of AKI in developed settings, in data from previous studies from SSA, and in this study. (DOCX 126 kb)
Additional file 3: Table S3. Summary of AKI studies undertaken in sub-Sahara Africa (SSA) published from 1990–2015. (DOCX 171 kb)

## Abbreviations

AKD: Acute kidney disease
AKI: Acute kidney injury
AKIN: Acute kidney injury network
ART: Antiretroviral therapy
CKD: Chronic kidney disease
Com-AKI: Community-acquired acute kidney injury
DM: Diabetes mellitus
GE: Gastroenteritis
HIV: Human immunodeficiency virus
IQR: Interquartile range
ISN: International Society of Nephrology
KDIGO: Kidney Disease Improving Global Outcomes
MAKISt: Malawi Acute Kidney Injury Study
NKD: No kidney disease
NSAIDs: Non-steroidal anti-inflammatory drugs
QECH: Queen Elizabeth Central Hospital
RRT: Renal replacement therapy
SSA: Sub-Sahara Africa
STOP: Sepsis and hypoperfusion, toxins, obstruction, parenchymal kidney disease
TB: Tuberculosis
TDF ART: Tenofovir based antiretroviral therapy
TDF: Tenofovir
