# Supplementary material for: Incidence, aetiology and outcome of community-acquired acute kidney injury in medical admissions in Malawi
Source: BMC Nephrol. 2017 Jan 14;18:21. doi: 10.1186/s12882-017-0446-4 (PMC5237521; doi:10.1186/s12882-017-0446-4)
Supplement: Additional file 1: Table S1. — Definitions – Acute Kidney Injury (AKI), Acute Kidney Disease/Disorder (AKD) without AKI, Chronic Kidney Disease (CKD) and No Kidney Disease (NKD). (DOCX 94 kb) [file 12882_2017_446_MOESM1_ESM.docx]

| **Acute Kidney Injury (AKI)** | | |
| --- | --- | --- |
|  | Creatinine Criteria* | Urine Output criteria* |
| Stage 1 | 1·5-1·9 times baseline**/***  OR  ≥26·5μmol/l increase**** | <0·5ml/kg/h for 6-12 hours |
| Stage 2 | 2·0-2·9 times baseline | <0·5ml/kg/h for ≥12 hours |
| Stage 3 | 3·0 times baseline  OR  Increase in creatinine to ≥353·6μmol/l*****  OR  Initiation of RRT | < 0·3ml/kg/h for ≥24 hours  OR  Anuria for ≥12 hours |
| *basis of diagnosis can be with either creatinine criteria or urine output criteria, with most advanced stage on either determining maximum stage  **baseline creatinine = lowest creatinine within last year; if not known, creatinine baseline estimated assuming eGFR of 75ml/min per 1·73 m2 (via MDRD equation), or taken as lowest creatinine during hospital stay (whichever lower)  ***known or assumed to have occurred within last 7 days  ****within 48 hours  *****must also fulfill at least stage 1 criteria | | |
| **Acute Kidney Disease/Disorder (AKD) without AKI*** | | |
| GFR <60 ml/min per 1·73 m^2^ for <3 months**  OR  Decrease in GFR by ≥35% or increase in SCr by >50% for <3 months** | | |
| *not fulfilling AKI criteria  **no biochemical (previous creatinine) or structural (kidneys <9cm bilaterally) evidence kidney damage >3 months old | | |
| **Chronic Kidney Disease (CKD)** | | |
| GFR <60 ml/min per 1·73 m^2^ for >3 months* | | |
| *biochemical (previous creatinine >3 months old) or structural (kidneys <9cm bilaterally) kidney damage >3 months old | | |
| **No Kidney Disease (NKD)** | | |
| GFR by ≥60ml/min per 1·73 m^2^, stable serum creatinine, and no evidence structural kidney damage >3 months old* | | |
| *includes patients with serum creatinine within reference range at screening, and patients with creatinine outside reference range at screening but who did not subsequently fulfill definition of AKI, AKD or CKD | | |
