# Supplementary material for: Incidence, aetiology and outcome of community-acquired acute kidney injury in medical admissions in Malawi
Source: BMC Nephrol. 2017 Jan 14;18:21. doi: 10.1186/s12882-017-0446-4 (PMC5237521; doi:10.1186/s12882-017-0446-4)
Supplement: Additional file 2: Table S2. — Comparison of AKI in developed settings, in data from previous studies from SSA, and in this study. (DOCX 126 kb) [file 12882_2017_446_MOESM2_ESM.docx]

|  | **Developed settings** | **Previous studies SSA** | **MAKIST** |
| --- | --- | --- | --- |
| **AKI incidence during inpatient stay** | Pooled incidence 21%^1^ | - | - |
| **Com-AKI vs. hAKI** | hAKI predominates | Com-AKI predominates^2,3^ | - |
| **Incidence of Com-AKI** | 1-4·3%^4,5^ | *4·5-15·2% in medical admissions^6,7^  16-24% in studies just focusing sepsis^89^ | 17·2% |
| **AKI severity** | 80% stage 1^1^  11% of AKI required RRT^1^ | *Stage 3 46-48%^6,8^ | Stage 1 21·6%  Stage 2 17·7%  Stage 3 60·8%  15% of AKI required RRT |
| **Patient demographics: age** | Median age 64 (US)^10^ | Mean age 28·7-44·4^11^ | Median age 41 |
| **Patient demographics: sex** | 35·3%M (US)^10^ | 56%M 1990-09; 52%M 2010-14^11^ | 60% M |
| **Patient demographics: co-morbidities** | HIV 0·5%  HTN 13·6%; DM 11·7%^12^  CCF 32%; CKD 31%^12^ | HIV 61% (Uganda)^8^  HTN 11·8%; DM 7·9% (Cameroon)^13^ | HIV 58·8%  HTN 15·7%; DM 6·5% |
| **Predominant cause of AKI: specialty** | Medical and surgical | Medical^2^ | - |
| **Predominant category of AKI (STOP)** | Sepsis and hypoperfusion | **Sepsis and hypoperfusion 57%^11^  Toxin: 14%^11^ | Sepsis and hypoperfusion 86·3% |
| **Predominant causes of AKI: specific** | Sepsis and complex surgery (trauma, cardiovascular surgery)^1^ | Infection: 53% of all medical causes^11^ | Infection: 59%  GE, TB and Malaria commonest  Toxin 3·3% as primary cause AKI but contributed to 75% AKI overall |
| **Access to RRT** | Free at point of delivery to all (UK) | ***17%^11^ | Free at point of delivery to all |
| **Inpatient mortality** | 21% across all stages^1^  Stage 3: 42%  Stage 3 undergoing RRT: 46% | 32% across all stages^11^  Stage 3 undergoing RRT 30% | 44·4% across all stages  Stage 3 49·5%  Stage 3 undergoing RRT 25% |
| **Renal recovery** | 10·3% persistent dysfunction^1^ | 45% (any recovery, adult survivors)^11^  16% persistent dysfunction at discharge | 47·2% (any recovery, all AKI)  63·2% persistent dysfunction at discharge |
| *prospective studies not just reporting cases requiring RRT  **percentage of medical causes  ***studies since 2010 | | | |
| SSA – sub Sahara Africa; AKI – acute kidney injury; com-AKI – community-acquired AKI; hAKI – hospital-acquired AKI; RRT – renal replacement therapy; HIV – human immunodeficiency virus; HTN – hypertension; DM – diabetes mellitus; CCF – congestive cardiac failure; CKD – chronic kidney disease; GE – gastroenteritis; TB - tuberculosis | | | |

Table S2: Comparison of AKI in developed settings, in data from previous studies from SSA, and in this study (MAKIST)

**References:**

1. Mehta RL, Cerdá J, Burdmann EA, Tonelli M, García-García G, Jha V, et al. International Society of Nephrology’s 0by25 initiative for acute kidney injury (zero preventable deaths by 2025): a human rights case for nephrology. Lancet Lond Engl. 2015 Jun 27;385(9987):2616–43.

2. Jha V, Parameswaran S. Community-acquired acute kidney injury in tropical countries. Nat Rev Nephrol. 2013 May;9(5):278–90.

3. Mehta RL, Burdmann EA, Cerdá J, Feehally J, Finkelstein F, García-García G, et al. Recognition and management of acute kidney injury in the International Society of Nephrology 0by25 Global Snapshot: a multinational cross-sectional study. Lancet Lond Engl. 2016 May 14;387(10032):2017–25.

4. Kaufman J, Dhakal M, Patel B, Hamburger R. Community-acquired acute renal failure. Am J Kidney Dis Off J Natl Kidney Found. 1991 Feb;17(2):191–8.

5. Wonnacott A, Meran S, Amphlett B, Talabani B, Phillips A. Epidemiology and outcomes in community-acquired versus hospital-acquired AKI. Clin J Am Soc Nephrol CJASN. 2014 Jun 6;9(6):1007–14.

6. Riley S, Diro E, Batchelor P, Abebe A, Amsalu A, Tadesse Y, et al. Renal impairment among acute hospital admissions in a rural Ethiopian hospital. Nephrol Carlton Vic. 2013 Feb;18(2):92–6.

7. El Hadji FK, Diouf B, Niang A, Ndiaye MF, Diop TM. Acute renal failure in adults in dakar. Saudi J Kidney Dis Transplant Off Publ Saudi Cent Organ Transplant Saudi Arab. 1999 Dec;10(4):513–4.

8. Bagasha P, Nakwagala F, Kwizera A, Ssekasanvu E, Kalyesubula R. Acute kidney injury among adult patients with sepsis in a low-income country: clinical patterns and short-term outcomes. BMC Nephrol. 2015;16:4.

9. Rayner BL, Willcox PA, Pascoe MD. Acute renal failure in community-acquired bacteraemia. Nephron. 1990;54(1):32–5.

10. Zeng X, McMahon GM, Brunelli SM, Bates DW, Waikar SS. Incidence, outcomes, and comparisons across definitions of AKI in hospitalized individuals. Clin J Am Soc Nephrol CJASN. 2014 Jan;9(1):12–20.

11. Olowu WA, Niang A, Osafo C, Ashuntantang G, Arogundade FA, Porter J, et al. Outcomes of acute kidney injury in children and adults in sub-Saharan Africa: a systematic review. Lancet Glob Health. 2016 Apr;4(4):e242–50.

12. Liangos O, Wald R, O’Bell JW, Price L, Pereira BJ, Jaber BL. Epidemiology and Outcomes of Acute Renal Failure in Hospitalized Patients: A National Survey. Clin J Am Soc Nephrol. 2006 Jan 1;1(1):43–51.

13. Kaze FF, Ekokobe FE, Halle MP, Fouda H, Menanga AP, Ashuntantang G. The clinical pattern of renal diseases in the nephrology in-patient unit of the Yaounde General Hospital in Cameroon: a five-year audit. Pan Afr Med J [Internet]. 2015 Jul 20 [cited 2016 Apr 1];21. Available from: http://www.ncbi.nlm.nih.gov/pmc/articles/PMC4575702/
