# Supplementary material for: Incidence, aetiology and outcome of community-acquired acute kidney injury in medical admissions in Malawi
Source: BMC Nephrol. 2017 Jan 14;18:21. doi: 10.1186/s12882-017-0446-4 (PMC5237521; doi:10.1186/s12882-017-0446-4)
Supplement: Additional file 3: Table S3. — Summary of AKI studies undertaken in sub-Sahara Africa (SSA) published from 1990–2015. (DOCX 171 kb) [file 12882_2017_446_MOESM3_ESM.docx]

| **Study reference and location** | **AKI Definition** | **Study overview: design** | | | **Study overview: patient number** | **Study overview: demographics** | | **Results: AKI incidence** | **Results: AKI Causes** | **Results: AKI Severity** | **Results: AKI Outcomes** | | **Other findings** |
| --- | --- | --- | --- | --- | --- | --- | --- | --- | --- | --- | --- | --- | --- |
|  |  | **Retro/Prosp** | **Patient cohort** | **Timeframe** | **Total no. AKI** | **Age (mean)** | **%M** |  |  |  | **Mortality** | **Renal Recovery** |  |
| Rayner *et al*, 1990^1^  Cape Town, South Africa (single centre, single cause) | Doubling or more of serum creatinine | Prosp | Patients admitted with community-acquired bacteraemia | 1 year | 58 |  |  | 24% | Sepsis (all) |  | 53% (22% if no ARF) |  |  |
| Bamgboye *et al,* 1993^2^  Lagos, Nigeria  (single centre) | RIFLE  Dialysis requiring | Retro | Patients with ARF treated at a dialysis centre | 1981-92 | 175 |  | 50·9% |  | Sepsis 38%, obstetric 26%, haemorrhage 10%, obstructive uropathy 3%, acute GN 5%, ‘holy water’ 3%, other nephrotoxins 5% |  | 39% | 52·6% | Many could not afford more than 1 dialysis session |
| Zewdu, 1994^3^  Addis Ababa, Ethiopia (single centre) |  | Retro | Patients with ARF treated at a renal unit | 1989-92 | 136 | 26·9W; 40·7M | 22·1% |  | Septic abortion 52%, Malaria 21%, Nephrotoxins 9% | 86% required dialysis | 33·8% |  | Highest mortality with malaria and obstetric complications |
| Mate-Kole *et al*, 1996^4^  Accra, Ghana (single centre) | Clinical  Dialysis requiring | Retro | Patients with ARF who underwent HD | 1972-92 | 170 | 32·5 | 51% |  |  |  | 32% |  | Highest mortality in obstetric patients |
| El Hadji *et al*, 1999^5^  Dakar, Senegal (2 centres) |  | Prosp | Patients referred to a medical clinic and an ICU with ARF | 1994-95 | 30 | 32 | 20% | 4·5% of medical admissions | Obstetric 50% (retroplacental haematoma 66·7%, eclampsia 20%, other: abortion, peripartum haemorrhage)  Medical 36% (malaria 55%)  Surgical 14% |  |  |  |  |
| Mtika *et al*, 2002^6^  Lilongwe, Malawi (single centre) | Dialysis requiring | Retro | Patients requiring HD | 1998-00 | 21 |  | 50% |  |  |  |  |  |  |
| Luyckx *et al*, 2005^7^  Johannesburg, South Africa (single centre, single cause) | elevated serum urea and creatinine above normal range, persistent oligoanuria, worsening renal function with time, or need for dialysis | Retro | Patients with ARF secondary to folk remedy use |  | 78 |  |  |  | Pre-renal 27%, ATN 27%, Hepatorenal Syndrome 6%, UTI 8%, Primary renal disease 8% |  | 41% |  | Higher if HIV vs. non-HIV |
| Bah *et al,* 2006^8^  Conakry University Hospital, Mali (single centre) | Acute increase in creatinine | Retro | All renal admissions | 1999-03 | 46 | 44 | 60·2% |  |  |  | 21% |  | 60% ‘had consumed decoctions of leaves and roots’ prior to presentation  14% had been to health centre |
| Kaballo *et al,* 2007^9^  Khartoum, Sudan (single centre) | Sudden rise in creatinine to > 3mg/dL in patients who had no history of previous renal impairment and who then required renal replacement | Retro | Patients who underwent dialysis for ARF | 2003-04 | 89 | 39 | 64% |  | 37% ATN secondary to volume depletion, infections (inc. malaria and typhoid fever), or snakebites  13% ATN secondary to paraphenylene-diamine (hair dye) suicide attempts  12% obstructive uropathy  9% glomerular disease |  | 18% | 60% |  |
| Arogundade *et al,* 2007^10^  Osun State, Nigeria (single centre) | Rapid increase in urea >10mmol/L; increase creatinine > 260umol/L + clinical  Dialysis requiring | Prosp | Patients undergoing renal replacement (HD and PD) for ARF | 1998-02 | 46 | 38·2 | 73·9% |  | Gastroenteritis 36·9%  Sepsis 30·5% |  | 43·5% |  | No difference in outcome PD vs. HD |
| El Imam *et al*, 2009^11^  Gezira Hospital, Sudan (single centre, single cause) | On ‘the basis of clinical and laboratory findings’ | Retro | Patients admitted to a renal unit with ARF secondary to Rift Valley Fever (RVF) | 2007-08 | 94 | 34 | 75% |  |  | 90% of ARF required RRT (peritoneal dialysis) | 31% |  |  |
| Friedericksen *et al*, 2009^12^  Cape Town, South Africa (single centre) | Serum creatinine level >177 µmol/l, or a twofold increase in baseline serum creatinine | Retro | Patients with ARF presenting to ICU | 2003-04 | 46 | 44·4 | 60% | 23% | ATN 82%, acute GN 8·6%, malignant HTN 6·5%, vasculitis 2·2% |  | 49% (vs. 17% if no ARF) |  | 6·5% HIV |
| Eghan *et al*, 2009^13^  Kumasi, Ghana (single centre) | Dialysis requiring | Retro | Patients undergoing HD at a new unit | 2006-07 | 6 | 28·7 | 33% |  |  |  |  |  |  |
| Lengani *et al,* 2010^14^  Burkina Faso (single centre) | Acute onset renal impairment, creatinine >240umol/L | Retro | Patients admitted with ARF | 2004-06 | 121 | 38·6 | 62% | 18·4% (of medical admissions) | Pre-renal 36%  Renal 47%  Obstruction 12% |  | 24% |  | Co-morbidities:  Heart failure 11%  HTN 11%  Liver failure 7%  Malignancy 3%  10% NSAIDS and 21·5% herbs pre hospital  Dialysis provided in 14/84 (17%) |
| Okpechi *et al,* 2011^15^  Cape Town, South Africa (single centre) | Acute presentation associated with renal impairment | Retro | All native kidney biopsies undertaken | 2000-10 | 274 | 36·8 | 45·2% | 21·3% of all biopsies were for ARF | Acute tubular necrosis 23·4%  HIVAN 15·0%  Lupus nephritis 14·2%  Acute interstitial nephritis 6·6%  Hypertensive nephroangiosclerosis 6·6%  Crescentic GN 5·1%  Post-infectious GN 4·4%  Diabetic nephropathy 4·0%  Mesangial proliferative GN 3·3%  Mesangiocapillary GN 2·9%  IgAN 1·8%  Chronic interstitial nephritis 1·5% |  |  |  |  |
| Arendse *et al*, 2011^16^  Cape Town, South Africa (single centre) | Patients requiring acute HD | Retro | HIV positive patients who underwent HD | 2002-07 | 117 | 34 (median) | 53·5% |  | ATN (58%) |  | 41% | 33% | 100% HIV  93·2% black |
| Ekrikpo *et al,* 2011^17^  Uyo, Nigeria (single centre) | Dialysis requiring | Retro | Patients undergoing HD | 2008-09 | 14 |  | 57% |  |  |  |  |  | Main predictor of mortality inability to pay for HD |
| Emem-Chioma et al, 2012^18^  Port Harcourt, Nigeria (single centre) | ADQI/RIFLE  Dialysis requiring | Retro | Patients undergoing HD for AKI | 1996-99 and 2007-09 | 62 | 41·3 | 55% | 1·9% admissions | Medical 71%  Sepsis 22·7%, acute GN 20·5%, GE 16%, toxinx 11·4% | 51% of AKI required HD | 43·5% |  |  |
| Chijioke *et al,* 2012^19^  Ilorin, Nigeria (single centre) | RIFLE  Dialysis requiring | Retro | Patients who underwent HD for ARF | 1989-09 | 138 | 29·4 | 42% |  | Sepsis 36%  Acute GN 16%  Septic abortion 12%  Toxin 11%  GE 8%  Post partum haemorrhage 8%  Drug 4%  Eclampsia 2%  Other 7% |  | 13·3% |  | Mean number HD sessions 2·24  Mean LOS 20·1 days |
| Okunola *et al*, 2012 and 2013^20,21^  Osogbo, Nigeria (single centre) | AKIN  Dialysis requiring | Retro | Patients who underwent HD for AKI | 2005-10 | 45 | 33·7 | 53% |  | Sepsis 36%, Obstetric related (pre-eclampsia, septic abortion, post-partum haemorrhage) 27%, Nephrotoxins 18%, Diarrhoea 9%, Acute GN 7%, Snake bite 2%, Rhabdomyolysis (burns) 2% |  | 28·8% |  | Major cause of mortality pre-eclampsia |
| Kilonzo *et al*, 2012^22^  [see also Carter *et al,* 2012^23^ and Callegari *et al*, 2013^24^]  Moshi, Tanzania (single centre) | Renal impairment requiring dialysis and history/clinical investigations suggestive of AKI | Retro | Patients who underwent PD for AKI | 2009-11 | 16 adults (4 children) | 33·9 | 56% |  | ATN 45%  GN 25%  Eclampsia/HELLP 10%  HUS 5%  Rhabdomyolysis 5% |  | 20% |  | 20% used traditional medicaiotns |
| Vachiat *et al,* 2013^25^  Johannesburg, South Africa (single centre) | RIFLE | Retro | HIV patients presenting with renal failure | 2005-06 | 78 | 37 (vs· 45 if HIV NR AKI) | 56% |  | Sepsis 63% (43% if HIV NR)  Haemodynamic instability 17%  Toxin 6%  Obstruction 5% |  | 44% (vs. 47% if HIV NR) |  |  |
| Phillips *et al,* 2013^26^  Ethiopia (3 centres – 1 urban, 2 rural) |  | Prosp | Investigation of AKI risk factor assessment in medical, surgical and obstetric inpatients |  |  |  |  |  | On medical wards infection commonest reason for admission (in particular TB and other infections related to HIV) |  |  |  | Assessment of:  Renal function 51-100%  Urine output 6-20%  Urine dip 30-57%  BP 61-98%  Assessment of risk factors for AKI lacking in up to 23% |
| Riley *et al,* 2013^27^  University of Gondar Hospital, Ethiopia (single centre; rural) | AKIN | Prosp | All admissions to hospital (not all screened) | 6/52 (July-August 2012) | 23 | 49·6 (vs· 41 if no AKI) | 61% | 15·2% | Sepsis (inc malaria) 35%, Cardiac disease 35%, Obstetric related 10%, Primary renal disease (pyelonephritis, nephrotic syndrome, post-strep GN, renal vein thrombosis) 20% | Stage 1 30%  Stage 2 22%  Stage 3 48% | 8·2% (vs· 7·8% if no renal impairment) | 38% persistent renal injury at discharge | 68% of total population had renal function measured – prevalence renal impairment 19·8%  LOS 11 days (AKI = no AKI) |
| Skinner *et al,* 2014^28^  Durban, South Africa (single centre) | RIFLE | Retro | Patients admitted to trauma ICU with AKI | 2008-11 | 102 | 39 |  | 15% |  |  | 57% |  | AKI independent predictor of mortality |
| Tshamba *et al*, 2014^29^  Lumbashi, DRC (single centre) | Dialysis requiring | Prosp | Random selection of patients undergoing HD | 2012 | 18 |  |  | 34% of patients on HD |  |  |  |  | Association of death with interruption in treatment (due to finances) |
| Okaka *et al,* 2014^30^  Benin, Nigeria (single centre) | Dialysis requiring | Retro | Patients undergoing HD | 2004-11 | 241 | 38·9M 33·1F | 60·9% | 19% of patients on HD | Sepsis commonest cause |  |  |  |  |
| Oluyombo et al, 2014^31^  Ido-Ekiti, Nigeria (single centre) | Dialysis requiring | Retro | Patients undergoing HD | 2010-13 | 26 | 38·1 | 67% | 14·8% of patients on HD |  |  |  |  |  |
| Bagasha *et al*, 2015^32^  Kampala, Uganda (single centre, single cause) | AKIN | Prosp | Patients admitted with sepsis to ER and medical wards (all screened) |  | 63 | 37 | 55·6% | 16% |  | Stage 1 29%  Stage 2 24%  Stage 3 46% | 21% | 59% persistent renal injury at 2/52 | Co-morbidity:  HIV 61%  HTN 4%  25% herbal medication prior to admission  No HD available |
| Kaze *et al*, 2015^33^  Yaounde, Cameroon (single centre) | RIFLE | Retro | Patients admitted to a renal unit | 2005-10 | 63 | 44·8 | 58·7% | 28% of renal admissions | ‘PARENCHYMAL’ 58·7%  -ATN 59% (malaria 36%, sepsis 23%, drugs 18·2%, eclampsia 13·6%, herbal remedy 9%)  -AIN 19% (drug induced 57%, infectious 29%, lymphoma 14%)  -Acute GN 11% (post-infective 100%)  -Vascular 11% (malignant HTN 50%, ANCA vasculitis 50%)  ‘FUNCTIONAL’ 25·4%  Enterocolitis 56%, Heart failure 31·3%, digestive haemorrhage 12·5%)  ‘OBSTRUCTIVE’ 15·9%  Prostate hypertrophy 60%, nephrolithiasis 20%, cervical cancer 20% |  |  |  | Co-morbidities:  HIV 15·9%  HTN 11·8%  DM 7·9% |

Table S3: Summary of AKI studies undertaken in sub-Sahara Africa (SSA) published from 1990-2015

**References:**

1. Rayner BL, Willcox PA, Pascoe MD. Acute renal failure in community-acquired bacteraemia. Nephron. 1990;54(1):32–5.

2. Bamgboye EL, Mabayoje MO, Odutola TA, Mabadeje AF. Acute renal failure at the Lagos University Teaching Hospital: a 10-year review. Ren Fail. 1993;15(1):77–80.

3. Zewdu W. Acute renal failure in Addis Abeba, Ethiopia: a prospective study of 136 patients. Ethiop Med J. 1994 Apr;32(2):79–87.

4. Mate-Kole MO, Yeboah ED, Affram RK, Ofori-Adjei D, Adu D. Hemodialysis in the treatment of acute renal failure in tropical Africa: a 20-year review at the Korle Bu Teaching Hospital, Accra. Ren Fail. 1996 May;18(3):517–24.

5. El Hadji FK, Diouf B, Niang A, Ndiaye MF, Diop TM. Acute renal failure in adults in dakar. Saudi J Kidney Dis Transplant Off Publ Saudi Cent Organ Transplant Saudi Arab. 1999 Dec;10(4):513–4.

6. Mtika VG, Muula AS, Chipolombwe J, Nyirongo J, Rajabu J. Renal replacement therapy at Lilongwe Central Hospital, Malawi. Trop Doct. 2002 Jul;32(3):163–5.

7. Luyckx VA, Steenkamp V, Stewart MJ. Acute renal failure associated with the use of traditional folk remedies in South Africa. Ren Fail. 2005;27(1):35–43.

8. Bah AO, Kaba ML, Diallo MB, Kake A, Balde MC, Keita K, et al. [Renal diseases--morbidity and mortality in Nephrology Service, National Hospital Donka]. Mali Méd. 2006;21(4):42–6.

9. Kaballo BG, Khogali MS, Khalifa EH, Khaiii EAG, Ei-Hassan AM, Abu-Aisha H. Patterns of ‘severe acute renal failure’ in a referral center in Sudan: excluding intensive care and major surgery patients. Saudi J Kidney Dis Transplant Off Publ Saudi Cent Organ Transplant Saudi Arab. 2007 Jun;18(2):220–5.

10. Arogundade FA, Sanusi AA, Okunola OO, Soyinka FO, Ojo OE, Akinsola A. Acute renal failure (ARF) in developing countries: which factors actually influence survival. Cent Afr J Med. 2007 Aug;53(5–8):34–9.

11. El Imam M, El Sabiq M, Omran M, Abdalkareem A, El Gaili Mohamed MA, Elbashir A, et al. Acute renal failure associated with the Rift Valley fever: a single center study. Saudi J Kidney Dis Transplant Off Publ Saudi Cent Organ Transplant Saudi Arab. 2009 Nov;20(6):1047–52.

12. Friedericksen DV, Van der Merwe L, Hattingh TL, Nel DG, Moosa MR. Acute renal failure in the medical ICU still predictive of high mortality. South Afr Med J Suid-Afr Tydskr Vir Geneeskd. 2009 Dec;99(12):873–5.

13. Eghan BA, Amoako-Atta K, Kankam CA, Nsiah-Asare A. Survival pattern of hemodialysis patients in Kumasi, Ghana: a summary of forty patients initiated on hemodialysis at a new hemodialysis unit. Hemodial Int Int Symp Home Hemodial. 2009 Oct;13(4):467–71.

14. Lengani A, Kargougou D, Fogazzi GB, Laville M. [Acute renal failure in Burkina Faso]. Néphrologie Thérapeutique. 2010 Feb;6(1):28–34.

15. Okpechi I, Swanepoel C, Duffield M, Mahala B, Wearne N, Alagbe S, et al. Patterns of renal disease in Cape Town South Africa: a 10-year review of a single-centre renal biopsy database. Nephrol Dial Transplant. 2011 Jan 6;26(6):1853–61.

16. Arendse C, Okpechi I, Swanepoel C. Acute dialysis in HIV-positive patients in Cape Town, South Africa. Nephrol Carlton Vic. 2011 Jan;16(1):39–44.

17. Ekrikpo UE, Udo AI, Ikpeme EE, Effa EE. Haemodialysis in an emerging centre in a developing country: a two year review and predictors of mortality. BMC Nephrol. 2011;12:50.

18. Emem-Chioma PC, Alasia DD, Wokoma FS. Clinical outcomes of dialysis-treated acute kidney injury patients at the university of port harcourt teaching hospital, Nigeria. ISRN Nephrol. 2013;2013:540526.

19. Chijioke A, Makusidi AM, Rafiu MO. Factors influencing hemodialysis and outcome in severe acute renal failure from Ilorin, Nigeria. Saudi J Kidney Dis Transplant Off Publ Saudi Cent Organ Transplant Saudi Arab. 2012 Mar;23(2):391–6.

20. Okunola Y, Ayodele O, Akinwusi P, Gbadegesin B, Oluyombo R. Haemodialysis practice in a resource-limited setting in the tropics. Ghana Med J. 2013 Mar;47(1):4–9.

21. Okunola OO, Ayodele OE, Adekanle AD. Acute kidney injury requiring hemodialysis in the tropics. Saudi J Kidney Dis Transplant Off Publ Saudi Cent Organ Transplant Saudi Arab. 2012 Nov;23(6):1315–9.

22. Kilonzo KG, Ghosh S, Temu SA, Maro V, Callegari J, Carter M, et al. Outcome of acute peritoneal dialysis in northern Tanzania. Perit Dial Int J Int Soc Perit Dial. 2012 Jun;32(3):261–6.

23. Carter M, Kilonzo K, Odiit A, Kalyesubula R, Kotanko P, Levin NW, et al. Acute peritoneal dialysis treatment programs for countries of the East African community. Blood Purif. 2012;33(1–3):149–52.

24. Callegari J, Antwi S, Wystrychowski G, Zukowska-Szczechowska E, Levin NW, Carter M. Peritoneal dialysis as a mode of treatment for acute kidney injury in sub-Saharan Africa. Blood Purif. 2013;36(3–4):226–30.

25. Vachiat AI, Musenge E, Wadee S, Naicker S. Renal failure in HIV-positive patients—a South African experience. Clin Kidney J. 2013 Dec;6(6):584–9.

26. Phillips LA, Allen N, Phillips B, Abera A, Diro E, Riley S, et al. Acute kidney injury risk factor recognition in three teaching hospitals in Ethiopia. South Afr Med J Suid-Afr Tydskr Vir Geneeskd. 2013 Jun;103(6):413–8.

27. Riley S, Diro E, Batchelor P, Abebe A, Amsalu A, Tadesse Y, et al. Renal impairment among acute hospital admissions in a rural Ethiopian hospital. Nephrol Carlton Vic. 2013 Feb;18(2):92–6.

28. Skinner DL, Hardcastle TC, Rodseth RN, Muckart DJJ. The incidence and outcomes of acute kidney injury amongst patients admitted to a level I trauma unit. Injury. 2014 Jan;45(1):259–64.

29. Tshamba HM, Van Caillie D, Nawej FN, Kapend FM, Kaj FM, Yav GD, et al. Risk of death and the economic accessibility at the dialysis therapy for the renal insufficient patients in Lubumbashi city, Democratic Republic of Congo. Pan Afr Med J [Internet]. 2014 Sep 23 [cited 2016 Apr 7];19. Available from: http://www.ncbi.nlm.nih.gov/pmc/articles/PMC4317069/

30. Okaka EI, Unuigbe EI. Eight year review of hemodialysis: treated patients in a tertiary center in Southern Nigeria. Ann Afr Med. 2014 Dec;13(4):221–5.

31. Oluyombo R, Okunola OO, Olanrewaju TO, Soje MO, Obajolowo OO, Ayorinde MA. Challenges of hemodialysis in a new renal care center: call for sustainability and improved outcome. Int J Nephrol Renov Dis. 2014;7:347–52.

32. Bagasha P, Nakwagala F, Kwizera A, Ssekasanvu E, Kalyesubula R. Acute kidney injury among adult patients with sepsis in a low-income country: clinical patterns and short-term outcomes. BMC Nephrol. 2015;16:4.

33. Kaze FF, Ekokobe FE, Halle MP, Fouda H, Menanga AP, Ashuntantang G. The clinical pattern of renal diseases in the nephrology in-patient unit of the Yaounde General Hospital in Cameroon: a five-year audit. Pan Afr Med J [Internet]. 2015 Jul 20 [cited 2016 Apr 1];21. Available from: http://www.ncbi.nlm.nih.gov/pmc/articles/PMC4575702/
